# Time-Course Analysis of Cyanobacterium Transcriptome: Detecting Oscillatory Genes

**DOI:** 10.1371/journal.pone.0026291

**Published:** 2011-10-18

**Authors:** Carla Layana, Luis Diambra

**Affiliations:** Centro Regional de Estudios Genómicos (CREG), Universidad Nacional de La Plata, Florencio Varela, Argentina; University of Georgia, United States of America

## Abstract

The microarray technique allows the simultaneous measurements of the expression levels of thousands of mRNAs. By mining these data one can identify the dynamics of the gene expression time series. The detection of genes that are periodically expressed is an important step that allows us to study the regulatory mechanisms associated with the circadian cycle. The problem of finding periodicity in biological time series poses many challenges. Such challenge occurs due to the fact that the observed time series usually exhibit non-idealities, such as noise, short length, outliers and unevenly sampled time points. Consequently, the method for finding periodicity should preferably be robust against such anomalies in the data. In this paper, we propose a general and robust procedure for identifying genes with a periodic signature at a given significance level. This identification method is based on autoregressive models and the information theory. By using simulated data we show that the suggested method is capable of identifying rhythmic profiles even in the presence of noise and when the number of data points is small. By recourse of our analysis, we uncover the circadian rhythmic patterns underlying the gene expression profiles from *Cyanobacterium Synechocystis*.

## Introduction

Physiological states of a living organism change as time goes by, forming a sequence of patterns that repeat themselves periodically or nearly periodically, such as the circadian rhythm. Circadian rhythms are endogenous self-sustaining oscillations that are regulated by a pacemaker composed of one or more biochemical oscillators [1]. These rhythms are observed in a wide variety of organisms, ranging from daily rhythms in photosynthesis in cyanobacteria to activity and sleep-wake cycles in rodents and humans. An important aspect of rhythmicity involves the control of specific target genes by oscillators that modulate and coordinate the transcription of genes governing key metabolic pathways. In this sense, it has been widely accepted that up to 10%–15% of all genes are expressed following a circadian rhythm [2]. In cyanobacteria, diverse activities such as cell division, amino acid uptake, nitrogen fixation, respiration, and carbohydrate synthesis are under circadian control [3], but a clear mechanistic link between physiological rhythms and the regulation of output genes is still lacking. Today many circadian-related genes have been explored using high-throughput DNA microarray technology [4]–[11]. Microarray experiments allow estimation of the relative expression of thousands of genes at each time point, and they are widely used for monitoring gene activities in a cell during biological processes. Based on microarray experiments, Schmitt et al. have reported 259 genes of *Cyanobacterium Synechocystis* that are responsive to light stimulus [9]. Furthermore, Kucho et al. have identified 237 cycling genes (54 genes under stringent condition) [12]. Many of these genes are related to energy metabolism, photosynthesis and respiration.

Data produced in microarray experiments carry a high degree of stochastic variation, obscuring the periodic pattern. Furthermore, microarray experiments are expensive, limiting the number of data points in a time series expression profile. The identification of the circadian expression pattern in time series data is challenging, because the measured data are often non-ideal, and efficient algorithms are needed to extract as much information as possible. Based on time series data, various approaches have been proposed to identify periodically expressed genes. Wichert et al. [13] applied the traditional periodogram and Fisher's test, while Ahdesmaki et al. [14] implemented a robust periodicity test assuming non-Gaussian noise. Alternatively, Luan and Li [15] employed guide genes and constructed cubic B-spline-based periodic functions for modeling, whereas Lu et al. [16] employed up to the third harmonics to fit the data and proposed a periodic normal mixture model. De Lichtenberg et al. [17] compared several approaches. Interestingly, the mathematically more advanced methods seem not to achieve a better performance compared with the Fast Fourier Transform (FFT) method. Recently, Hughes et al., have introduced a non-parametric algorithm named JTK_CYCLE that is robust against outliers [18]. Alternatively, some authors have assumed the circadian signal to be a simple sinusoid. In this sense, McDonald and Rosbash performed cross-correlation coefficients between the experimental profiles and differently phased cosine waves [19]. Similarly, Kucho et al. applied the Cosinor method to the same data analyzed here [12]. Although these algorithms have their own advantages, they were all developed based on some assumptions. For example, a good spectral estimation needs several data points, and Cosinor assumes that the biological process follows a trigonometric function. However, in many real-world applications, such as microarray experiments, data are scarce and noisy, and the underlying dynamics not necessarily obeys a trigonometric function. Consequently, these methods may fail to detect genes with a periodical expression and produce artifacts (false positives).

To address this issue, a new computational technique for the identification of periodic patterns in relatively short time series is introduced. By combining the autoregressive model, maximum entropy methods and information theory concepts, a novel inference approach is proposed to effectively screen out periodically expressed genes. The technique was applied to simulated expression profiles and to experimental data. We found 164 genes that are periodically expressed in gene expression data sets from two independent cyanobacteria cultures [12]; 93 of them have not been reported before. Furthermore, by assuming that key circadian genes must conserve both the dynamics and the phase in the biological replicates, we were also able to identify genes of the core pacemarker circuit as well as essential genes for the photosynthesis, respiration and energy metabolism processes.

## Results

### Synthetic time series analysis

**Figure 1:**
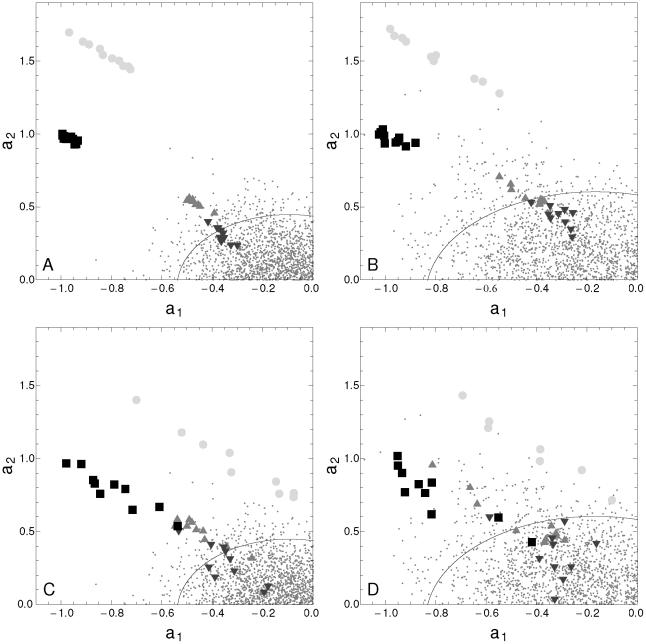
Dynamic paramters of simulated data. Scatter plot of the dynamic parameters 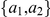 corresponding to 24-point time series (A), and 12-point time series (B). Dark gray down-triangles correspond to saw-teeth signals, gray up-triangles correspond to square-step signals, light gray circles correspond to sinusoidal signals of a 48 h period, and black squares to a sinusoidal signal of a 24 h period. The small gray points correspond to surrogate time series, the ellipsoid corresponds to the quantile contour at level 0.9. Open symbols represent time series contaminated with a low level of noise (standard deviation of 5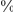 of the signal amplitude), while filled symbols represent time series contaminated with a high level of noise (standard deviation of 15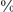). We also observe that symbols corresponding to each of the four oscillatory patterns (24-point time series) form distinguishable clusters at low noise. We can also observe that sampling frequency affects the characterization.

In order to calibrate our technique we analyzed four synthetic time series. Figure 1 depicts the scatter plot of the autoregressive coefficient 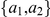 of each noisy signal: low noise in the top panels and high noise in the bottom panels. Results for 96 h time series length (24 data points) are summarized in the left panels, while the results for 48 h time series length (first 12 data points of the former case) are summarized in the right panels. We observed that the autoregressive coefficients corresponding to long time series at low noise (Fig. 1A) are clearly clustered forming four groups corresponding to the four patterns. Both the time series length (Fig. 1B) and noise level (Fig. 1C) impact on the intracluster dispersion. As expected, longer signals are more robust against noise effects. In addition, except for the saw-teeth signal (dark gray), almost all 96 h time series can be characterized as oscillatory at the confidence level of 0.90, while for 48 h time series (Fig. 1B) around 50% of cases are not classified as oscillatory at this level of confidence. Figs. 1B, 1C,1D illustrate that the ellipsoid quantile also increases with the time series length. The combined effect is a lower number of significant periodic signals.

**Figure 2:**
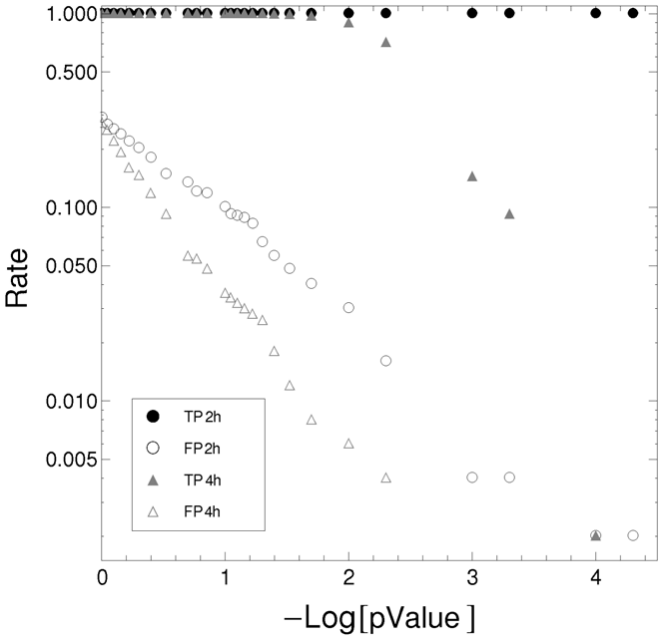
True positive rate and false positive rate. The true positive rate (filled symbols) decays after a significance level of 0.01. At this level the percentage of false positives is 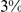 and 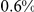 for 2 h and 4 h sampling rates, respectively. We use two sampling resolution: 2 h (circles) and 4 h (triangles).

Furthermore, we also noted that the sampling frequency impacts on the analysis, that better sampling (more data points per period) contributes to improving the characterization, in agreement with previous findings [27]. For example, 48 h period (light gray) time series are farther from the ellipsoidal quantile than 24 h period (black) time series. However, the cluster corresponding to the high sampling frequency (light gray circles) is less robust against noise than low sampling frequency (black squares). These results could suggest a guideline for the experimental design of microarray experiments to look for oscillatory genes. Figure 2 shows the true positive rate and the false positive rate, for two different sampling resolutions, as a function of -Log[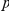-value]. Our method is highly sensitive and specific at the significance level of 0.01, even when the expression profiles are sampled at 4-hour intervals, as in the circadian microarray analyzed below. The example shown in Fig. 2 corresponds to sinusoidal signals of different periods corrupted with noise (standard deviation of 20% of amplitude). The sensitivity and specificity of the method depend on the sample rate, the period of the oscillation, the wave form, and the noise level, but not on the phase and amplitude.

### Cyanobacteria microarray analysis

**Figure 3:**
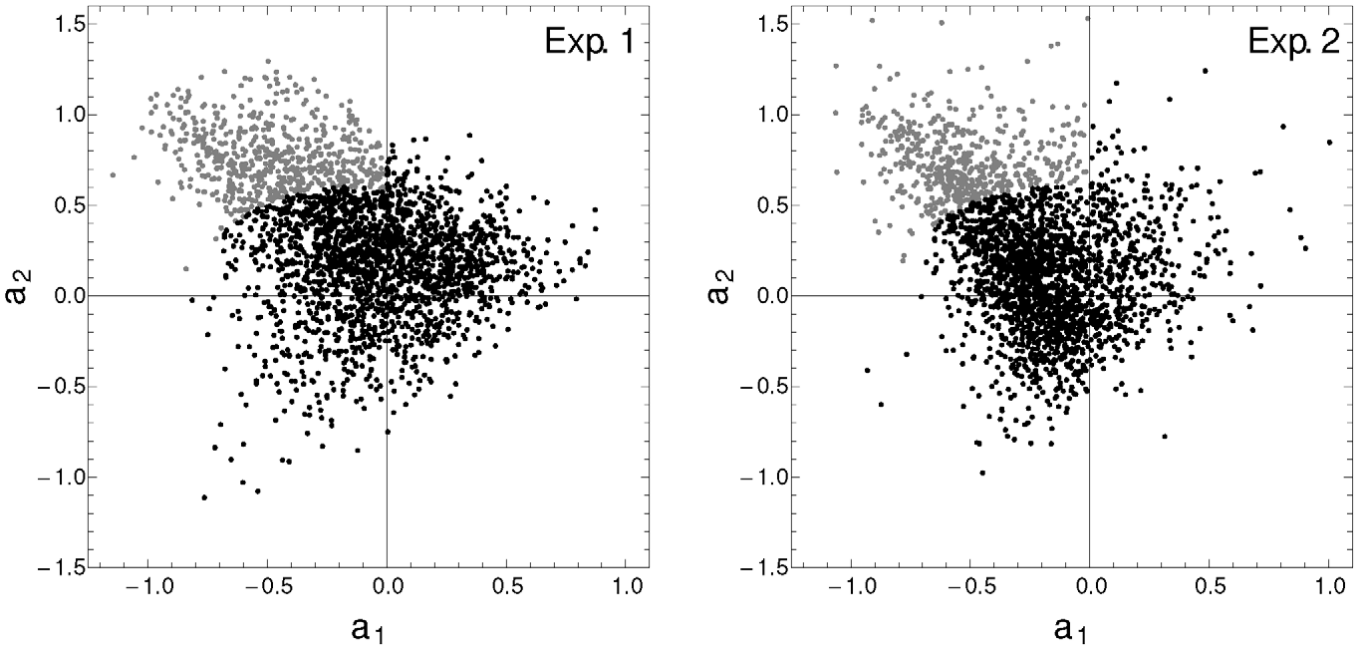
Dynamic paramters of microarray data. Scatter plot of the dynamic parameters 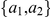 corresponding to each gene profile of Exp. 1 (A), and Exp. 2 (B). The parameter space was arbitrarily divided into two regions. Gray dots correspond to genes whose dynamic parameter values are 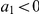 and 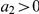 and are significant at the 0.9 level. The analysis reports 587 genes with oscillatory pattern from Exp. 1 and 514 from Exp. 2.

In Fig. 3 we show the autoregressive coefficients associated with the expression dynamics of 3070 genes of a cyanobacteria microarray. Fig. 3A corresponds to the first biological replicate (Exp. 1) and Fig. 3B corresponds to the second biological replicate (Exp. 2). Based on the previous study of synthetic time series, we select those genes whose autoregressive coefficients fall in the region 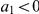 and 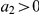, and that are outside the ellipsoidal quantile 0.9. At this significance level, Fig. 2 suggests the existence of 5% of false the scatter plot of the positive in sinusoidal profiles with 20% of noise. The analysis reveals 527 and 473 genes that have an oscillatory expression profile associated at this significant level in Exp. 1 and Exp. 2, respectively. Even though the biological replicates were synchronized simultaneously via photic training, only 164 of these genes are considered as oscillatory in both experiments. Table S1, in supplemental material, lists all these genes and their associated functions. Many of these genes have unknown function.

**Figure 4:**
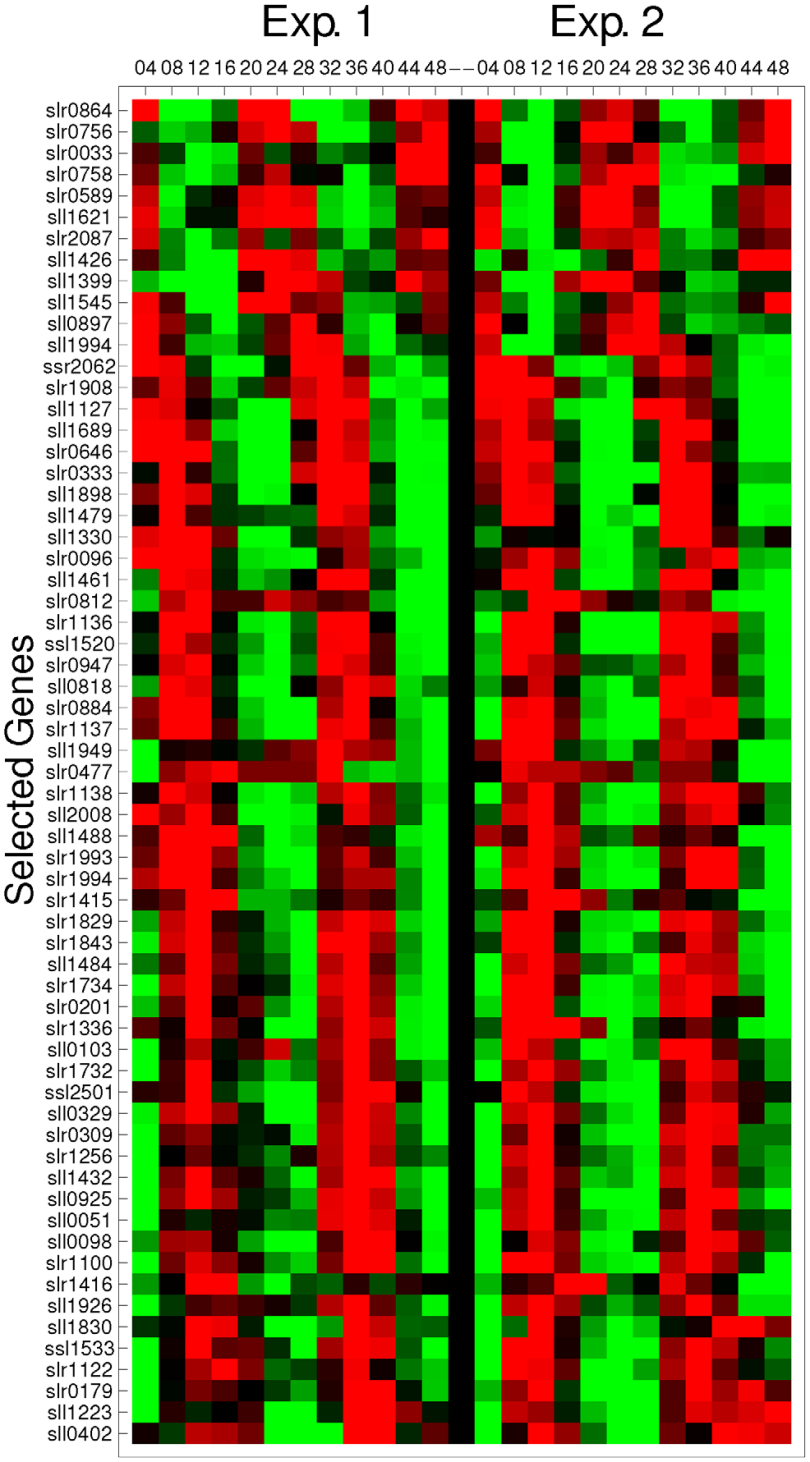
Expression profile of 63 genes with oscillatory patterns in both experiments. They are sorted by the phases of Exp. 1. The expression was normalized to the mean expression at all time points and represented by a gray scale.

After identifying the genes with an oscillatory expression, our aim is to identify genes that could be associated with the circadian clock machinery (CCM) that generates the circadian oscillations. In this sense we use the following hypotheses: (i) The expression profile must be circadian in both replicates; (ii) The dynamics of the CCM genes must be preserved through the two biological replicates; (iii) As replicates are synchronized, we expect that genes of CCM have a small phase shift between both replicates. With these working hypotheses, from the 164 oscillatory genes we select those whose model-based distance (Eq. 9) between replicates is shorter than 0.6 and the phase shift is smaller than 0.6 (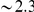 h). After this filtering step we identify 63 cycling genes. Fig. 4 depicts the expression profile of the 63 genes with an oscillatory pattern in both experiments. They are sorted by the phases of Exp. 1.

**Figure 5:**
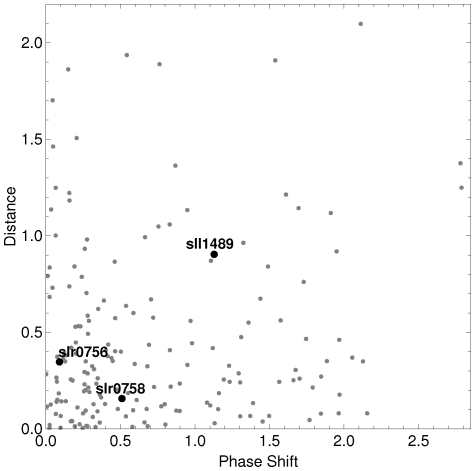
Scatter plot of the 63 selected genes. The horizontal axis corresponds to the phase shift between gene profiles from the experiments, while the vertical axis corresponds to the dynamic distance between gene profiles from the different experiments. Black dots correspond to three circadian clock genes. KaiA (slr0756) and KaiC (slr0758) genes have a similar phase and similar dynamics in both replicates.

**Figure 6:**
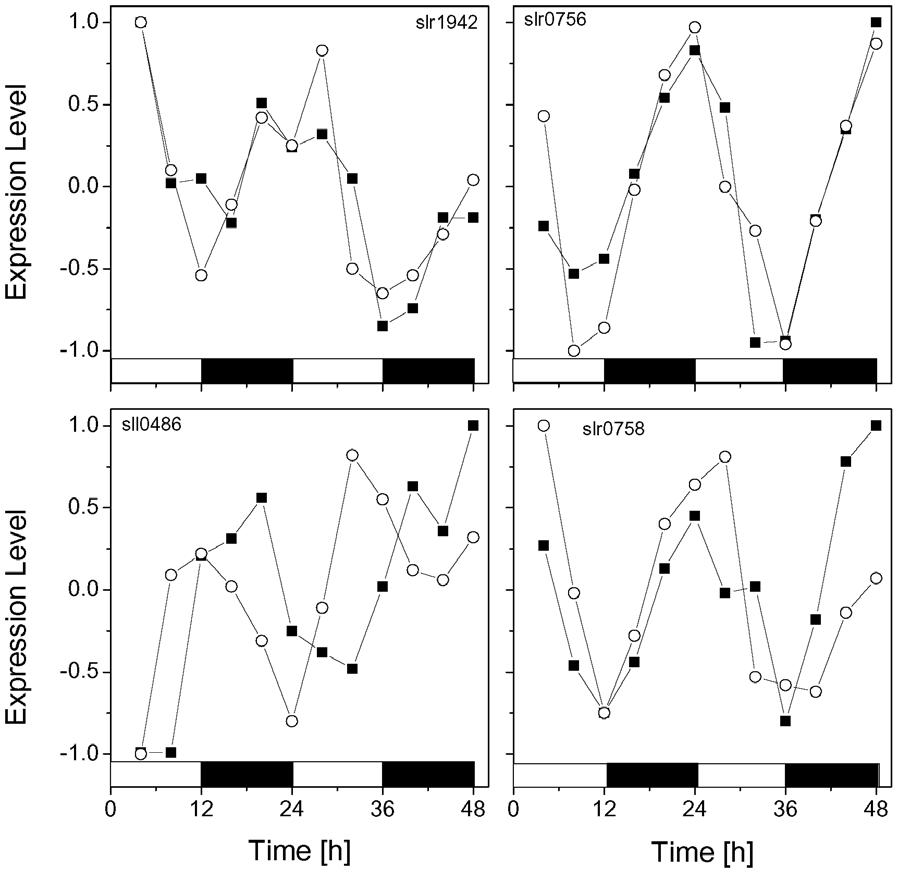
Expression profile of the four genes. Expression of kaiA (slr0756), kaiB3 (sll0486), kaiC1 (slr0758) and kaiC3 (slr1942), corresponding to the circadian clock machinery of cyanobacteria. Black squares correspond to Exp. 1, while circles correspond to Exp. 2.

The Synechocystis genome encodes one KaiA gene, three KaiB genes, and three KaiC genes [28]. Four of them, KaiA (slr0756), KaiB3 (sll0486), KaiC1 (slr0758) and KaiC3 (slr1942), were successfully measured in the experiments used here [12]. Fig. 5 depicts a scatter plot of the phase shift and the dynamic distance between the replicates of the 63 selected oscillatory genes. The black dots in Fig. 5 correspond to the genes slr0756 (KaiA) and slr0758 (KaiC1) that belong to the circadian clock of the cyanobacteria and were detected by the criteria. We want to remark that our criteria identify two circadian core genes, in contrast to the Cosinor based criteria [12] that only identify the slr0756 gene. Fig. 5 also shows that a circadian related gene sll1489, which is a circadian phase modifier CpmA homolog, is less synchronized between replicates than KaiA and KaiC1 genes. We identify that the dynamics associated with these genes is conserved between replicates, and slr0756 is highly synchronized by photic training. Genes sll0486 and slr1942 have oscillatory expression behavior at the confidence level of 0.90 in Exp. 2, but not in Exp 1. Fig. 6 shows the expression profile of the four Kai genes; black squares correspond to Exp. 1, while open circles correspond to Exp. 2. We can see that all these genes have an oscillatory pattern.

**Figure 7:**
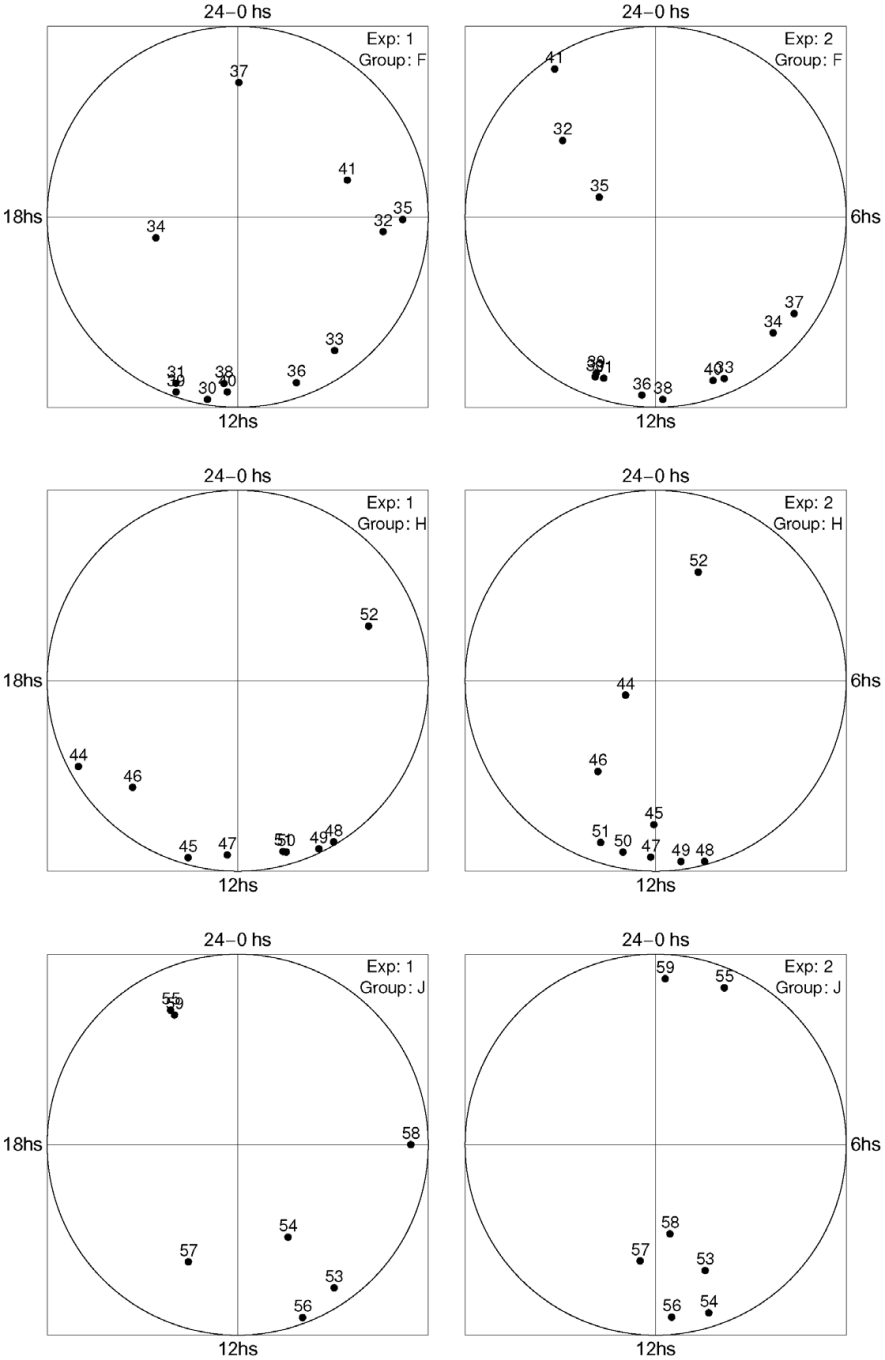
Phase diagrams of genes listed in **Table 1**. Group F corresponds to genes related to energy metabolism. Group H corresponds to genes related to photosynthesis and respiration. Group J corresponds to genes related to regulatory functions. 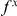 and 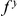 correspond to the horizontal axis and to the vertical axis, respectively (see details in the text).

In addition to the circadian clock genes, among the 63 genes with oscillatory pattern we found genes associated with other processes. Of particular interest are 28 genes linked to energy metabolism (Group F, 12 genes), photosynthesis and respiration (Group H, 9 genes), and regulatory functions (Group J, 7 genes). Table 1 lists the accession number, enzyme name and function (when available) (see Table S1, in supplemental material, for the list of 164 genes). Fig. 7 shows the phase diagrams of the gene expression profiles associated with these processes in both replicates. It is observed that many circadian genes are strongly synchronized between replicates. In group F (top panels) the genes sll0329 (30), sll1196 (31), sll1479 (33), slr0884 (36), slr1734 (38), slr1793 (39) and slr1843 (40) are synchronized between experiments. Furthermore, genes sll0329, sll1196 and slr1793 are almost co-expressed in both experiments. In group H (medium panels) all genes, except for slr2034 (52), are synchronized between experiments. The genes linked to cytochrome C, sll1889 (48), slr1136 (49), slr1137 (50) and slr1138 (51), are co-expressed in both experiments. In group J (bottom panels) genes sll1330 (53), slr0081 (54), slr0947 (56), slr1416 (57) and slr1983 (59) are synchronized between experiments and all, except the last one, are also co-expressed.

**Table 1:** Some genes with circadian expression.

Index	Accession	Enzyme Name	Function
30 F	sll0329*	6-phosphogluconate dehydrogenase	Pentose phosphate pathway
31 F	sll1196*	Phosphofructokinase	Glycolysis
32 F	sll1234#	Adenosylhomocysteinase	Amino acids and amines
33 F	sll1479*	6-phosphogluconolactonase	Pentose phosphate pathway
34 F	slr0301	Phosphoenolpyruvate synthase	Pyruvate metabolism
35 F	slr0394#	Phosphoglycerate kinase	Glycolysis
36 F	slr0884*,#	GAPDH 1	Glycolysis
37 F	slr1705	Aspartoacylase	Amino acids and amines
38 F	slr1734	G6PDN assembly protein	Pentose phosphate pathway
39 F	slr1793*	Transaldolase	Pentose phosphate pathway
40 F	slr1843*	Glucose 6-phosphate dehydrogenase (G6PDN)	Pentose phosphate pathway
41 F	slr2094	Fructose-1,6-/sedoheptulose-1,7-bisphosphatase	Other
44 H	sll0741	Pyruvate flavodoxin oxidoreductase	soluble electron carriers
45 H	sll1220*	Diaphorase su. of the bidirectional hydrogenase	Hydrogenase
46 H	sll1223	Diaphorase su. of the bidirectional hydrogenase	Hydrogenase
47 H	sll1484**	Type 2 NADH dehydrogenase	NADH dehydrogenase
48 H	sll1899*	Cytochrome c oxidase folding protein	Respiratory terminal oxidases
49 H	slr1136*	Cytochrome c oxidase su. II	Respiratory terminal oxidases
50 H	slr1137*	Cytochrome c oxidase su. I	Respiratory terminal oxidases
51 H	slr1138*	Cytochrome c oxidase su. III	Respiratory terminal oxidases
52 H	slr2034	Putative homolog of plant HCF136	Photosystem II
53 J	sll1330*	Two-component response regulator OmpR sf.	Regulatory functions
54 J	slr0081	Two-component response regulator OmpR sf.	Regulatory functions
55 J	slr0312**	Two-component response regulator NarL sf.	Regulatory functions
56 J	slr0947**	Response regulator for energy transfer	Regulatory functions
		from phycobilisomes to photosystems	
57 J	slr1416	similar to MorR protein	Regulatory functions
58 J	slr1738	Transcription regulator Fur family	Regulatory functions
59 J	slr1983**	Two-component hybrid sensor and regulator	Regulatory functions

Genes exhibiting circadian rhythm in both experiments that are linked to energy metabolism (F), to photosynthesis and respiration (H) and to regulatory functions (J). The functional categories of the genes are according to KEGG.

*Corresponds to genes detected by the cosinor method, and

**corresponds to genes detected by the cosinor method with relaxed filtering conditions reported by Kucho et al. 2004.

# Corresponds to genes whose expression is influenced by light reported by Stephanopoulos et al. 2004. su. denotes subunit, and sf. denotes subfamily.

## Discussion

We presented an alternative method for the identification of oscillatory genes in microarray time series gene expression data. This approach uses both autoregressive models and the MaxEnt approach to secure the actual periodic genes existing in a microarray data set. We have also used a dynamic-based distance between two temporal expression profiles to compare the dynamics between two replicates. We found that the dynamic features extracted by the modeling procedure of the core clock genes are a signature through biological replicates. For a fixed amount of data points, for example 12 data points as typical time series, our results suggest the following prescriptions: (i) at high noise level, to record two periods (48 h times series length) sampled every 4 h; (ii) at low noise level, to record one period (24 h times series length) sampled every 2 h.

A notable issue with the regulation of cyanobacterial metabolism is the circadian rhythm of many processes [29]. In particular, the operation of photosynthesis and respiration are under circadian control [30], and the coordinate regulation of these two processes is an essential determinant of the overall energy balance. Our analysis shows that many gene components of the respiratory electron transport chain were cycling (Table 1). H group contains two genes that are related to the hydrogenase: HoxE (sll1220) and HoxU (sll1223). In a previous analysis [12] only the gene HoxE was found to be circadian. In agreement with Kucho's analysis, we also found that slr1136, slr1137, slr1138, sll1899 and sll1484 have circadian behavior. Furthermore we also identified sll0741 (pyruvate flavodoxin oxidoreductase) and slr2034 (putative homolog of plant HCF136), which have not previously been reported as cycling genes. Group F contains genes related to energy metabolism that have been previously identified as circadian [9], [12]. Our analysis suggests that two pentose pathway enzymes (sll0329 and slr1793) are co-expressed in both experiments with a phosphofructokinase (sll1196). In Group J we found that four response regulators containing a DNA-binding domain (sll1330, slr0081, slr0312 and slr0947) [31] were expressed with circadian rhythm. Summing up, the present analysis identified 164 genes as cycling, 93 of them have not been reported before. Many of these genes are expected to be circadian. However, 42 of these 93 genes, with unknown function, can be associated to circadian processes.

In conclusion, we propose that our procedure is a promising statistical tool for finding oscillatory expressed genes of any period in a microarray data set. The code of our procedure is freely available, as a Mathematica script, upon request.

## Materials and Methods

### Modeling Gene Expression Dynamics and MaxEnt Approach

We also used MaxEnt approach and information theory (IT) [20], [21] concepts for modeling the dynamics of the gene expression and defined a model-based distance between two dynamics [22]. In this sense, we assumed that the processes generating the time series 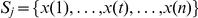, associated with a gene 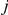, could be approximated by an autoregressive model of order 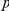, say AR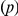, as follows
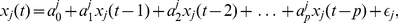
(1)where 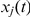 denotes the expression of gene 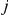 at time 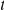. The elements of the vector 
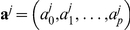
 are the autoregressive coefficients (AC) and 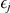 is a random value, which is assumed to have a Gaussian distribution, with null expected value and variance 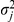. It is important to note in this context that AR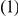 processes cannot capture periodic patterns except for alternations with period two. However, AR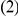 processes are able to generate periodic dynamics when AC satisfy 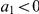 and 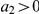. AR models of higher order imply more AC to estimate; as one expects to deal with short and noisy time series, here we will consider only AR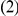. However, the method can be generalized to higher orders. The corresponding autoregressive coefficients 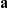 can be determined using the MaxEnt approach from the gene expression time series 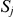 of 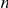 values. The essential idea of MaxEnt is to formally treat and quantify our ignorance [23] in order to provide the most parsimonious inference method, using all available data and avoiding the introduction of additional hypotheses. We will describe the procedure considering only one gene profile, since the modeling procedure of each gene is independent of each other. In order to infer AC consistent with the model and data, we shall assume that each set of AC 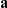 is realized with probability 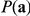. In other words, we introduce a normalized probability distribution; over the collection of conceivable sets of 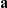, this is the essential IT ingredient. Thus, the expectation values 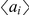 are defined as
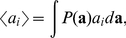
(2)and the relative entropy [20], [21] associated with the probability distribution is given by
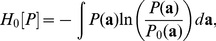
(3)where 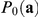 is an appropriately chosen *a priori* distribution. Following the central tenets MEP [20], [21], the entropy (3) is to be maximized, subject to the normalization conditions and the constraints (2) [24]. In practice, these constraints are enforced by introducing Lagrange multipliers,
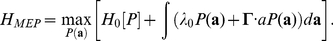
(4)A variation of Eq. (4) with respect to 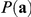 gives
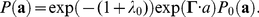
(5)When 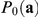 is proportional to 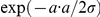, the probability distribution, 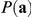, is a Gaussian centered in 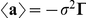, *i.e.*,
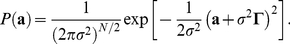
(6)


Now, the idea is to interpret the data set 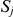 according to Eq. (1) in the following manner 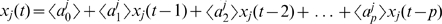 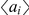. This allows the elimination of the Lagrange multipliers 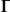 and the expression of the most probable set 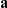, compatible with the constraints, solely in terms of the expression levels
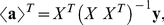
(7)where 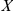 is the regression matrix whose 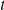-th row is 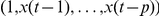 for 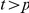, and 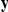 is the vector 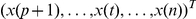. Once the AR coefficients are determined, the next step is to classify the behavior associated with expression profiles that satisfy 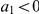 and 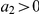 as oscillatory. In contrast to other methods (Cosinor, JTK_CYCLE), the present method does not determine the period, the phase and the amplitude of oscillations. To overcome this limitation, we determine the position of cycling genes in a phase diagram, by computing the cross-correlation between the expression profile with a sine time series 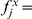
Corr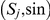, and a cosine time series 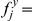
Corr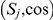. In a phase diagram, the phase of profile 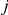, 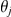, is given by 
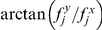
 and corresponds to the angular coordinate. The distance to the center point of the circle is given by 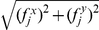, this distance corresponds to the radial coordinate and assesses how well the expression profile 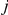 fits a sinusoidal function. Thus, two genes with similar angular coordinates are synchronized; while genes near to unitary circle are associated to sinusoidal form.

After modeling we will focus our attention on the model based distance between two dynamics. From the information theory viewpoint, the amount of uncertainty of the probability distribution is measured by the entropy. Associated with the entropy function there is a divergence measure, also known as Kullback-Leibler distance [20], between 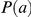 and 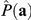, which will be denoted here by 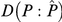


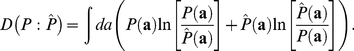
(8)In particular, for two distributions (6), 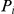 and 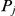, associated with the dynamics of two gene expression profiles 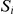 and 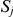, respectively, the model-based distance is
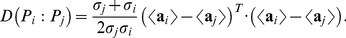
(9)This divergence measure is positive, definite, symmetric, and fulfills the triangular inequality.

### Significance Analysis

In identifying periodic genes, the analysis of significance becomes mandatory after AC estimation. The usual approach consists in specifying a well-defined null hypothesis. The second step is to compute the AC of this process from original data. Finally, we test the null hypothesis against the observations. In this article, our null hypothesis is that the time series corresponding to a given profile is not periodic, i.e., that the associated AC do not satisfy 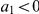 and 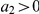. This null hypothesis is obtained by randomly shuffling the original data point. With this operation, the new time series loses the temporal order but preserves its statistical distribution properties. Then, we estimate the distribution of AC for an ensemble of surrogate data sets, which are only different realizations of the hypothesized stochastic process. Then, rather than estimating error bars on the AC of the original data, we calculate the ellipsoid quantile at the given confidence level from the 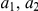 values corresponding to the surrogate data sets as natural generalizations of the multivariate quantile considered by Chaudhuri [25]. In this paper we considered as significant rhythms the time series whose associated AC values are out of the ellipsoid quantile, at the confidence level of 0.90, from the surrogate data sets. We worked with an ensemble of 10000 surrogate time series for each case.

### Experimental Data

Kucho et al. [12] monitored genome-wide mRNA levels, for 3,070 *Cyanobacterium Synechocystis* chromosomal genes simultaneously, over two circadian cycle periods (48 h), at 4 h intervals. RNA samples were isolated from two independent cyanobacterial cultures. Each RNA sample was used for three independent microarray experiments. Thus, a maximum of six data points per gene was obtained for each time point (i.e., three measurements for each biological replicate). Each biological replicate was treated independently with the same procedure until the final step of the cycling genes' characterization of their rhythmicity and phase. Spots meeting any of the following criteria were flagged and not used for the analysis: (i) the GenePix Pro did not find the spot area automatically, (ii) the net signal intensity was 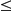
0, (iii) the percentage of saturated pixels in the spot area was 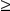
25, and (iv) severe noise was present. Genes carrying more than one unflagged data point at any time point were removed from our analysis. The data are available from KEGG database [26]. In a previous analysis of these data using the Cosinor method, 237 genes that exhibited circadian rhythms were identified [12]. We normalized each gene expression time series to mean zero and a maximum of 1.0, as is sometimes recommended when measurements are not on a comparable scale.

As simulated data we constructed four synthetic time series, corresponding to different oscillatory patterns that are 96 h long and were sampled every 4 h (see Fig. S1). The time course A (dark gray) corresponds to a saw-teeth signal, B (gray) corresponds to a square-step signal, both having a 24 h period. The other two signals, C and D, are sinusoidal and the corresponding periods are 48 h (light gray) and 24 h (black), respectively. These time series were contaminated with 10 realizations of additive Gaussian noise at two different levels (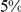 of the signal amplitude, denoted by low noise, and 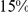 of the signal amplitude, denoted by high noise). Additionally, we also tested the sensitivity and specificity of the method to detect cycling behavior at two different sampling resolutions (2 h and 4 h). For each sampling resolution, we have generated a test set of 1000 expression profiles across 2 full days. The period lengths were uniformly distributed between 20 h and 28 h, and the phase was uniformly distributed across the entire cycle. Half of the transcripts were entirely random (normally distributed), while the amplitudes of the remaining cycling expression profiles were uniformly distributed between 1 and 6. Each data point was added to a standard normal random variable to simulate experimental noise (mean zero, standard deviation of 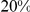 of the profile amplitude). In all cases, the noise and short length are two important features that these synthetic signals share with the signals obtained from microarrays.

## Supporting Information

Figure S1 **Four synthetic oscillatory time series used to simulate expression profiles.** A (dark gray down-triangle) is a saw-teeth signal, B (grey up-triangle) is a square-step signal, C (light grey circle) and D (black square) are sinusoidal time series. A, B and D have a 24 h period, while C has a 48 h period. Each signal was contaminated with 10 realization of additive noise to generate 10 noisy time series, which were used to test the performance of the method to identify oscillatory behavior in short and noisy time series.
(EPS)

Table S1 **All oscillatory genes found.** 164 genes exhibiting cycling behavior in both experiments and the functional categories of the according to KEGG categories (http://genome.kazusa.or.jp/cyanobase/Synechocystis/genes/category.txt).
(PDF)
