# Supplementary material for: Time-Course Analysis of Cyanobacterium Transcriptome: Detecting Oscillatory Genes
Source: PLoS One. 2011 Oct 18;6(10):e26291. doi: 10.1371/journal.pone.0026291 (PMC3196541; doi:10.1371/journal.pone.0026291)
Supplement: Table S1 — All oscillatory genes found. 164 genes exhibiting cycling behavior in both experiments and the functional categories of the according to KEGG categories (http://genome.kazusa.or.jp/cyanobase/Synechocystis/genes/category.txt). (PDF) [file pone.0026291.s002.pdf]

1	sll0402	aspartate aminotransferase	A	Amino acid biosynthesis	Aspartate family
2	sll0450	cytochrome b subunit of nitric oxide reductase	A	Amino acid biosynthesis	Glutamate family / Nitrogen assimilation
3	sll1502	NADH-dependent glutamate synthase large subunit	A	Amino acid biosynthesis	Glutamate family / Nitrogen assimilation
4	sll1981	acetolactate synthase	A	Amino acid biosynthesis	Branched chain family
5	slr0550	dihydrodipicolinate synthase	A	Amino acid biosynthesis	Aspartate family
6	slr0898#	ferredoxin--nitrite reductase	A	Amino acid biosynthesis	Glutamate family / Nitrogen assimilation
7	slr2130	3-dehydroquinate synthase	A	Amino acid biosynthesis	Aromatic amino acid family
8	sll0892	aspartate 1-decarboxylase	B	Biosynthesis of cofactors, prosthetic groups, and carriers	Pantothenate
9	sll1127*	1,4-dihydroxy-2-naphthoate synthase	B	Biosynthesis of cofactors, prosthetic groups, and carriers	Menaquinone and ubiquinone
10	sll1545**	glutathione S-transferase	B	Biosynthesis of cofactors, prosthetic groups, and carriers	Thioredoxin, glutaredoxin, and glutathione
11	sll1994#	porphobilinogen synthase (5-aminolevulinate dehydratase)	B	Biosynthesis of cofactors, prosthetic groups, and carriers	Cobalamin, heme, phycobilin and porphyrin
12	slr0477	phosphoribosylglycinamide formyltransferase	B	Biosynthesis of cofactors, prosthetic groups, and carriers	Purine ribonucleotide biosynthesis
13	slr0633*	thiamine biosynthesis protein ThiG	B	Biosynthesis of cofactors, prosthetic groups, and carriers	Thiamin
14	slr1239	pyridine nucleotide transhydrogenase alpha subunit	B	Biosynthesis of cofactors, prosthetic groups, and carriers	Nicotinate and nicotinamide
15	sll1550	probable porin; major outer membrane protein	C	Cell envelope	Membranes, lipoproteins, and porins
16	slr0646*	probable D-alanyl-D-alanine carboxypeptidase	C	Cell envelope	Murein sacculus and peptidoglycan
17	slr0809	dTDP-glucose 4,6-dehydratase	C	Cell envelope	Surface polysaccharides, lipopolysaccharides and antigens
18	slr0985	dTDP-4-dehydrorhamnose 3,5-epimerase	C	Cell envelope	Surface polysaccharides, lipopolysaccharides and antigens
19	slr1272	probable porin; major outer membrane protein	C	Cell envelope	Membranes, lipoproteins, and porins
20	slr1615#	perosamine synthetase	C	Cell envelope	Surface polysaccharides, lipopolysaccharides and antigens
21	slr1908	probable porin; major outer membrane protein	C	Cell envelope	Membranes, lipoproteins, and porins
22	slr1933	dTDP-4-dehydrorhamnose 3,5-epimerase	C	Cell envelope	Surface polysaccharides, lipopolysaccharides and antigens
23	sll0040	positive phototaxis protein, homologous to chemotaxis protein CheW	D	Cellular processes	Chemotaxis
24	sll0041	phytochrome-like photoreceptor protein for positive phototaxis; homologous to methyl-accepting chemotaxis protein	D	Cellular processes	Chemotaxis
25	sll0897	DnaJ protein, heat shock protein 40, molecular chaperone	D	Cellular processes	Chaperones
26	sll0420	urease beta subunit	E	Central intermediary metabolism	Other
27	sll0643	urease accessory protein G	E	Central intermediary metabolism	Other
28	slr1256**	urease gamma subunit	E	Central intermediary metabolism	Other
29	slr1367	glycogen phosphorylase	E	Central intermediary metabolism	Polysaccharides and glycoproteins
30	sll0329*	6-phosphogluconate dehydrogenase	F	Energy metabolism	Pentose phosphate pathway
31	sll1196*	phosphofructokinase	F	Energy metabolism	Glycolysis
32	sll1234#	adenosylhomocysteinase	F	Energy metabolism	Amino acids and amines
33	sll1479*	6-phosphogluconolactonase	F	Energy metabolism	Pentose phosphate pathway
34	slr0301	phosphoenolpyruvate synthase	F	Energy metabolism	Pyruvate and acetyl-CoA metabolism
35	slr0394#	phosphoglycerate kinase	F	Energy metabolism	Glycolysis
36	slr0884*#	glyceraldehyde 3-phosphate dehydrogenase 1 (NAD+)	F	Energy metabolism	Glycolysis
37	slr1705	aspartoacylase	F	Energy metabolism	Amino acids and amines
38	slr1734*	glucose 6-phosphate dehydrogenase assembly protein	F	Energy metabolism	Pentose phosphate pathway
39	slr1793*	transaldolase	F	Energy metabolism	Pentose phosphate pathway
40	slr1843*	glucose 6-phosphate dehydrogenase	F	Energy metabolism	Pentose phosphate pathway
41	slr2094	fructose-1,6-/ sedoheptulose-1,7-bisphosphatase	F	Energy metabolism	Other
42	slr1993*	PHA-specific beta-ketothiolase	G	Fatty acid, phospholipid and sterol metabolism	
43	slr1994	PHA-specific acetoacetyl-CoA reductase	G	Fatty acid, phospholipid and sterol metabolism	
44	sll0741	pyruvate flavodoxin oxidoreductase	H	Photosynthesis and respiration	Soluble electron carriers
45	sll1220*	putative diaphorase subunit of the bidirectional hydrogenase	H	Photosynthesis and respiration	Hydrogenase
46	sll1223	diaphorase subunit of the bidirectional hydrogenase	H	Photosynthesis and respiration	Hydrogenase
47	sll1484**	type 2 NADH dehydrogenase	H	Photosynthesis and respiration	NADH dehydrogenase
48	sll1899*	cytochrome c oxidase folding protein	H	Photosynthesis and respiration	Respiratory terminal oxidases
49	slr1136*	cytochrome c oxidase subunit II	H	Photosynthesis and respiration	Respiratory terminal oxidases
50	slr1137*	cytochrome c oxidase subunit I	H	Photosynthesis and respiration	Respiratory terminal oxidases
51	slr1138*	cytochrome c oxidase subunit III	H	Photosynthesis and respiration	Respiratory terminal oxidases
52	slr2034	putative homolog of plant HCF136	H	Photosynthesis and respiration	Photosystem II
53	sll1330*	two-component system response regulator OmpR subfamily	J	Regulatory functions	
54	slr0081	two-component response regulator OmpR subfamily	J	Regulatory functions	
55	slr0312**	two-component response regulator NarL subfamily	J	Regulatory functions	
56	slr0947**	response regulator for energy transfer from phycobilisomes to photosystems	J	Regulatory functions	
57	slr1416	similar to MorR protein	J	Regulatory functions	
58	slr1738	transcription regulator Fur family	J	Regulatory functions	
59	slr1983**	two-component hybrid sensor and regulator	J	Regulatory functions	
60	sll0306	RNA polymerase group 2 sigma factor	K2	DNA replicatio and transcription	-
61	sll0377	transcription-repair coupling factor	K2	DNA replicatio and transcription	
62	sll0865	excinuclease ABC subunit C	K2	DNA replicatio and transcription	
63	sll1572**	DNA polymerase III alpha subunit [Contains: Ssp dnaE intein]	K2	DNA replicatio and transcription	
64	sll1689*	group2 RNA polymerase sigma factor SigE	K2	DNA replicatio and transcription	-
65	slr0346	ribonuclease III	K2	DNA replicatio and transcription	-
66	slr0965	DNA polymerase III beta subunit	K2	DNA replicatio and transcription	
67	sll0830	elongation factor EF-G	M	Translation	Protein modification and translation factors
68	sll2008	processing protease	M	Translation	Degradation of proteins, peptides, and glycopeptides
69	slr0033	glutamyl-tRNA(Gln) amidotransferase subunit C	M	Translation	Aminoacyl tRNA synthetases and tRNA modification
70	slr1549	polypeptide deformylase	M	Translation	Protein modification and translation factors
71	slr1751	periplasmic carboxyl-terminal protease	M	Translation	Degradation of proteins, peptides, and glycopeptides
72	sll0385	ATP-binding protein of ABC transporter	N	Transport and binding proteins	
73	sll0681	phosphate transport system permease protein PstC homolog	N	Transport and binding proteins	
74	sll0855	putative channel transporter	N	Transport and binding proteins	
75	sll1104**	periplasmic substrate-binding protein of a TRAP-type permease that mediates sodium-dependent glutamate transport GtrC	N	Transport and binding proteins	
76	sll1864	probable chloride channel protein	N	Transport and binding proteins	
77	slr0096	low affinity sulfate transporter	N	Transport and binding proteins	
78	slr0797	cobalt-transporting P-type ATPase (cobalt efflux pump) involved in cobalt tolerance	N	Transport and binding proteins	
79	slr0864*	ATP-binding protein of ABC transporter	N	Transport and binding proteins	
80	slr1336**#	H+/Ca2+ exchanger	N	Transport and binding proteins	
81	sll0818**	tetrapyrrole methylase family protein	O	Other categories	Other
82	sll0992	putative esterase	O	Other categories	Other
83	sll1432*	putative hydrogenase expression/ formation protein HypB	O	Other categories	Hydrogenase
84	sll1489	circadian phase modifier CpmA homolog	O	Other categories	Other
85	sll1621*	AhpC/TSA family protein	O	Other categories	Other
86	slr0201	heterodisulfide reductase subunit B	O	Other categories	Other
87	slr0309**	probable methyltransferase	O	Other categories	Other
88	slr0756*	circadian clock protein KaiA homolog	O	Other categories	Other
89	slr0758**	circadian clock protein KaiC homolog	O	Other categories	Other
90	slr1063**#	probable glycosyltransferase	O	Other categories	Other
91	slr1085*	probable glycosyltransferase	O	Other categories	Other
92	slr1302	protein involved in constitutive low affinity CO2 uptake	O	Other categories	Other
93	slr2087**	c-type cytochrome biogenesis protein Ccs1	O	Other categories	Other
94	slr2136**	GcpE protein homolog	O	Other categories	Other
95	sll0051**	hypothetical protein	P	Hypothetical	
96	sll0072	hypothetical protein	P	Hypothetical	
97	sll0098**	hypothetical protein	P	Hypothetical	
98	sll0103#	hypothetical protein	P	Hypothetical	
99	sll0253	hypothetical protein	P	Hypothetical	
100	sll0804**	hypothetical protein	P	Hypothetical	
101	sll0925**	hypothetical protein	P	Hypothetical	
102	sll1119	hypothetical protein	P	Hypothetical	
103	sll1247**	hypothetical protein	P	Hypothetical	
104	sll1358*	putative oxalate decarboxylase, periplasmic protein	P	Hypothetical	
105	sll1399	hypothetical protein	P	Hypothetical	
106	sll1461*	hypothetical protein	P	Hypothetical	
107	sll1488*	hypothetical protein	P	Hypothetical	
108	sll1659	hypothetical protein	P	Hypothetical	
109	sll1898*	hypothetical protein	P	Hypothetical	
110	sll1926**	hypothetical protein	P	Hypothetical	
111	slr0013	hypothetical protein	P	Hypothetical	
112	slr0049	hypothetical protein	P	Hypothetical	
113	slr0142**	hypothetical protein	P	Hypothetical	
114	slr0147	hypothetical protein	P	Hypothetical	
115	slr0179	hypothetical protein	P	Hypothetical	
116	slr0244	hypothetical protein	P	Hypothetical	
117	slr0320	hypothetical protein	P	Hypothetical	
118	slr0517	hypothetical protein	P	Hypothetical	
119	slr0589*	hypothetical protein	P	Hypothetical	
120	slr0645**	hypothetical protein	P	Hypothetical	
121	slr0812	hypothetical protein	P	Hypothetical	
122	slr0888	hypothetical protein	P	Hypothetical	
123	slr0921#	hypothetical protein	P	Hypothetical	
124	slr0976	hypothetical protein	P	Hypothetical	
125	slr1100**	hypothetical protein	P	Hypothetical	
126	slr1122	hypothetical protein	P	Hypothetical	
127	slr1196	periplasmic protein, function unknown	P	Hypothetical	
128	slr1415	hypothetical protein	P	Hypothetical	
129	slr1461**	hypothetical protein	P	Hypothetical	
130	slr1732*	hypothetical protein	P	Hypothetical	
131	slr1896	hypothetical protein	P	Hypothetical	
132	ssl1046#	hypothetical protein	P	Hypothetical	
133	ssl1498	hypothetical protein	P	Hypothetical	
134	ssl1918	hypothetical protein	P	Hypothetical	
135	ssr0692#	hypothetical protein	P	Hypothetical	
136	ssr1256	hypothetical protein	P	Hypothetical	
137	ssr1951	hypothetical protein	P	Hypothetical	
138	ssr2062*	hypothetical protein	P	Hypothetical	
139	sll0588	unknown protein	Z	Unknown	
140	sll0733	unknown protein	Z	Unknown	
141	sll0922	unknown protein	Z	Unknown	
142	sll1359	unknown protein	Z	Unknown	
143	sll1426	unknown protein	Z	Unknown	
144	sll1515#	glutamine synthetase inactivating factor IF17	Z	Unknown	
145	sll1830	unknown protein	Z	Unknown	
146	sll1949	unknown protein	Z	Unknown	
147	sll1950**	unknown protein	Z	Unknown	
148	slr0226	unknown protein	Z	Unknown	
149	slr0333	unknown protein	Z	Unknown	
150	slr0582**#	unknown protein	Z	Unknown	
151	slr1073#	unknown protein	Z	Unknown	
152	slr1084	unknown protein	Z	Unknown	
153	slr1240	unknown protein	Z	Unknown	
154	slr1576**	unknown protein	Z	Unknown	
155	slr1667	hypothetical protein (target gene of sycrp1)	Z	Unknown	
156	slr1681	unknown protein	Z	Unknown	
157	slr1829*	putative poly(3-hydroxyalkanoate) synthase component	Z	Unknown	
158	slr1928	type 4 pilin-like protein	Z	Unknown	
159	ssl1520	unknown protein	Z	Unknown	
160	ssl1533**	unknown protein	Z	Unknown	
161	ssl2162	unknown protein	Z	Unknown	
162	ssl2501	unknown protein	Z	Unknown	
163	ssr1038	unknown protein	Z	Unknown	
164	ssr3465	unknown protein	Z	Unknown	
